# Health crisis within a crisis: Effect of COVID-19 on STI services for young adults in Lusaka, Zambia

**DOI:** 10.1371/journal.pgph.0004891

**Published:** 2025-07-03

**Authors:** Ganizani Mwale, Mpundu Makasa

**Affiliations:** 1 Health Services, University of Zambia, Lusaka, Zambia; 2 School of Public Health, University of Zambia, Lusaka, Zambia; University of Ghana, GHANA

## Abstract

In Zambia, approximately 5% of women and 8% of men aged 15–49 reported having a Sexually Transmitted Infection (STI) 12 months prior to the 2018 Zambia Demographic Health Survey. Notably, 62% of women and 73% of men who had an STI sought treatment at a clinic, signifying the importance attached to health services by STI treatment seekers. Regrettably, during the COVID-19 pandemic, entry points for accessing STI health services were closed as a public health measure to control the spread of infection. This study assessed the pandemic’s effect on accessibility, availability, and delivery of STI health services in Lusaka. An explanatory sequential mixed-methods approach, incorporating a retrospective record review over a period of two years and a hermeneutic phenomenological qualitative design, was used to explore the lived experiences of healthcare providers. We found that Out-Patient Department (OPD) attendance dropped by 23% and 31% during the first and second phases of the pandemic, respectively. There was a positive correlation (p = 0.002) between OPD attendance and reported STI cases. The lived experiences of health providers revealed challenges in availability of STI health services, stemming from a range of factors that included truncated service points, reduced working hours, and limited interactions, all of which affected STI diagnosis. Stay-at-home orders, fear, lockdowns, and logistical challenges impeded access to STI health services. We established an intricate nexus between COVID-19 and the accessibility, availability, and delivery of STI health services and products. We recommend addressing pandemic-induced barriers to individuals’ access to STI health services through enhanced health communication, adopting flexible service delivery models, adapting healthcare infrastructure, addressing health provider challenges, and investing in research and preparedness to guide future pandemic responses.

## Introduction

Sexually transmitted infections (STIs) have been observed for a significant amount of time. Despite public awareness, prevention strategies, and medical advances, STIs remain a public health problem [1]. The World Health Organisation [2] states that more than one million sexually transmitted infections are acquired every day worldwide. Annually, an estimated 374 million new infections of the four main STIs – chlamydia, gonorrhea, syphilis, and trichomoniasis– occur [2]. Sexually transmitted infections have consequences ranging from causing pelvic inflammatory disease, infertility, cervical cancer, and adverse birth outcomes.

The African region is disproportionately affected with new STI cases estimated at 96 million in 2021 [3]. Considering that STIs have a profound impact on the health and lives of populations, the Sustainable Development Goals (SDGs), as outlined in the 2030 Agenda for Sustainable Development [4], include a strategy that involves reducing STI cases as one of the key health targets. This strategy has faced several challenges due to the Corona Virus Disease 2019 (COVID-19) pandemic in 2020 and 2021 that diverted health systems’ attention from other diseases [5]. The diverted attention might have led to a surge in STI cases with the potential to affect the attainment of SDGs on health. At present, sexually transmitted infections place a substantial burden on the budgets of national health systems in middle- and low- income countries [4].

The COVID-19 pandemic disrupted healthcare provision including access to STI services globally as evidenced by different studies. In the United Kingdom (UK) for instance, the pandemic led to the closure of specialist clinics resulting in limited access to STI health services [6]. This was further noticed in the United States of America (USA) with disruptions in access to STI health services observed at both state and national levels [7]. The impact of COVID-19 was not confined to specific regions, as studies from South Africa also indicated reduced access to contraception, maternity care, and HIV treatment [8].

In Zambia 5% of women and 8% of men aged 15–49 reported having had an STI or symptoms of an STI in the 12 months prior to the 2018 Zambia Demographic Heath Survey. It was striking that 62% of women and 73% of men who had an STI or symptoms of an STI sought treatment at health facilities [9]. This suggests that access to health services in the treatment of STIs is essential for Zambians. All health facilities in the country utilize syndromic management of STIs, which involves grouping diseases with similar symptoms and administering treatment using drugs that cover the most common causes of those symptoms.

Zambia’s already strained health system was further burdened when the country recorded its first COVID-19 case in March 2020, traced to international travelers 14 days after their arrival [10]. By the end of April, the number of suspected and admitted COVID-19 cases had increased to overwhelming levels, exerting pressure on a health system already burdened with other conditions like STIs, HIV, and Malaria [10]. Despite not imposing lockdowns like neighboring countries, the Ministry of Health launched the National Strategy for Reducing New Infections of COVID-19 to raise community awareness about the importance of complying with public health measures [11]. Furthermore, to reduce the spread of COVID-19, the Ministry of Health through Statutory Instrument No. 22 of 2020 placed limits on public gatherings and adjusted working hours allowing employees to work from home, thereby promoting social distancing [12].

Healthcare services were scaled down, with several services at health facilities completely discontinued to make way for the establishment of COVID-19 screening and admission centers. Health services like Antiretroviral Therapy (ART) created alternative ways of providing medication to patients in the form of six-moth multi-month dispensation (6MMD) in anticipation of the increase in COVID-19 cases coupled with the need to avoid patient exposure to COVID-19 [13]. However, other health services lacked alternative mechanisms to ensure continuity of care at health facilities.

Zambia has a high percentage of sexually active young adults who engage in risky sexual behaviors, placing them at increased risk of STIs and HIV [14]. Yang et al. [15] postulate that 72.2% of participants in their Zambian study reported having engaged in risky sexual experiences. The STI testing coverage in Zambia is estimated at 43.4% [16]. Coupled with the outbreak of the COVID-19 pandemic, this coverage may have reduced further. The pandemic introduced new dynamics in access to health services, such as the closure of adolescent and young adult hubs. These closures led to a 33% reduction in the number of adolescents and young people (AYP) accessing sexual and reproductive health services and products, including STI screening at the hubs [17].

The limited or non-availability of STI services and products to young adults who are already underserved might have led to increased STIs and HIV incidence reversing progress made towards the 2030, 9595-95 targets (whereby 95% of people living with HIV (PL-HIV) should be diagnosed, 95% of those diagnosed with HIV should be receiving antiretroviral therapy (ART), and 95% of all those receiving ART should achieve viral suppression (VLS)) [16 and 17]. This study aimed to examine the effect that the COVID-19 pandemic on the accessibility, availability, and delivery of STI health services and products to young adults.

## Methods

### Study design

The study employed a mixed-methods explanatory sequential design to explore the accessibility, availability, and delivery of STI health services and products to young adults. The quantitative phase utilized a retrospective record review (RRR). Secondary, de-identified quantitative data were collected from OPD registers between 2019 and 2021, using SmartCare system and epidemiological registers. The qualitative phase adopted a hermeneutic phenomenological design to investigate the lived experiences of healthcare providers before and during the COVID-19 pandemic. Qualitative data were collected through in-depth interviews with healthcare providers.

### Setting and sample

The study was conducted in Chelstone sub-district, one of the seven sub-districts of Lusaka. With a population of 498,016, Chelstone sub-district is designated as zone one. It comprises seven (7) clinics, one level 1 hospital and one hospice. The health facilities provide a range of services to the community, including maternal and newborn care, as well as prevention and treatment of communicable and non-communicable diseases. Chelstone sub-district was purposively selected for this study due to the high concentration of tertiary and higher education institutions that enroll large numbers of young adults.

The sample for the quantitative phase comprised young adults (15–49yrs) who visited health facilities between 1^st^ June 2019 – 31^st^ May 2021. A census of secondary data was conducted using general OPD attendance records, filtered by sex, age group, month, year, and recorded STI cases during the study period.

The qualitative phase involved interviews with clinicians who worked in OPD settings before, during, and after the COVID-19 pandemic. Clinician recruitment was based on the inclusion criteria requiring that participants had worked in OPD during the COVID-19 pandemic period (2020 – 2021) and prior to the pandemic in 2019. Efforts were made to achieve gender balance in the selection process. Recruitment took place between 20^th^ December 2022 and 15^th^ March 2023.

### Data collection

Quantitative secondary data were collected from Out-Patient Department (OPD) registers at seven (7) health facilities (six clinics and one hospital) in the Chelstone Sub-District that provided STI and COVID-19 services during the study period. A census of general OPD attendance records was conducted, with data filtered by sex, age group, month, year, and reported STI cases between 1^st^ June 2019 and 31^st^ May 2021. Additionally, daily epidemiological data on COVID-19 cases were collected for the period 1^st^ March 2020 – 31^st^ May 2021. Medical records were accessed from SmartCare database between 15^th^ November 2022 and 15^th^ December 2022. The OPD registers did not contain individually identifiable patient information. The data were categorized into three eight-month phases (timeframe) covering the period from 1st June 2019 – 31st May 2021. These were defined as the pre-COVID-19 phase, the first phase and the second phase of the COVID-19 pandemic.

The qualitative phase involved interviews with clinicians who had worked in OPD settings before, during, and after the COVID-19 pandemic. Written informed consent was obtained prior to the in-depth interviews, which were conducted by the principal researcher from 20^th^ December 2022 to 15^th^ March 2023. All interviews were conducted in English using a semi-structured interview guide (S1 Data) and lasted between one hour to one and half hours. Interviews were audio-recorded and transcribed verbatim. The sample size for the qualitative phase was determined through data saturation.

### Variables

In this study, the concept of availability referred to the ease with which healthcare providers and services could be reached or utilized. Accessibility was defined as the ability of individuals to obtain STI services during the COVID-19 period. Delivery encompassed the healthcare providers’ capacity to effectively administer services and products to patients. To frame the discussion, the study employed Levesque’s Conceptual Framework, with a specific emphasis on the dimensions of availability and accommodation.

### Rigor

To enhance the rigor of the qualitative phase, three strategies were adopted. First, to ensure credibility and confirmability, member checking was employed. This involved each participant verifying accuracy of their verbatim transcript, allowing for the confirmation of completeness and correctness [18]. Second, to promote reflexivity, prolonged engagement and the maintenance of a reflexive journal were employed throughout the study. Prolonged engagement provided contextual understanding of OPD operations while reflexive notes supported critical reflection during the discussion of findings with the co-researcher. Third, to achieve dependability, triangulation was applied converging data from interviews, field notes and quantitative records.

### Data analysis

To analyze the quantitative data, descriptive statistics – including mean, median, and standard deviation – were calculated for the variables: gender, OPD attendance, STI cases, and COVID-19 cases before and during the pandemic. Further analysis was conducted to determine the relationships between OPD attendance and COVID-19 pandemic, and between OPD attendance and STI cases. Segmented regression analysis was performed using OPD attendance as the proxy dependent variable and the three time periods as the independent variables. Differences between periods were examined using Kruskal-Walli’s test. The quantitative findings informed the development of interview questions for the qualitative phase of the study.

For qualitative data analysis, two researchers independently conducted thematic coding of the verbatim transcripts using the web-based version of Atlas.ti, following an initial phase of data familiarization and note-taking. The researchers inductively developed codes (S2 Data) by assigning labels to segments of text that reflected meaningful concepts related to the study objectives, ideas, and emerging patterns. The generated codes were then compared and discussed; similarities were identified and consolidated into 12 subthemes, which were subsequently grouped into three overarching themes. Code comparison between researchers enhanced the credibility and trustworthiness of the findings.

### Ethics statement

Ethical clearance was obtained from the University of Zambia Biomedical Research Ethics Committee (UNZABREC; Ref. No. 3191–2022) and the National Health Research Authority (Ref. No. NHRA0000018/31/10/2022). All quantitative data collected from OPD registers were de-identified and did not contain any information that could lead to the identification of individuals. Data were extracted from the SmartCare system using filters for age group, sex, diagnosis, month, and year.

All clinical healthcare providers received an information sheet outlining the background and purpose of the study. Recruitment was conducted from 20^th^ December 2022 to 15^th^ March 2023. Written informed consent was obtained from each clinician prior to the in-depth interviews. Participants were informed of their right to withdraw from the interview at any point without consequences. They were also assured that withdrawal would not affect their employment in anyway. The study did not involve data collection from minors or child participants (defined as individuals 18 years and below).

## Results

### Quantitative analysis

#### OPD attendance.

Data analysis from the OPD registers (S3 Data) indicated that, across the seven health facilities in the sub-district, a total of 415,821 individuals visited the health facilities between 1^st^ June 2019 and 31^st^ May 2021, with an additional 178,515 recorded as revisits. The data further showed that among first-time OPD visits, 54% were females (n = 225,435) and 46% were males (n = 125,527), all between the ages 15 and 49 years. Among the revisits, 53% were females (n = 95,275) and 47% were males (n = 50,153) within the same age group.

#### STI, OPD attendance and COVID-19 Cases by Sex and Year.

The year-by-year data analysis is presented in Table 1. The data show that the average number of STI cases recorded in 2019 (seven months) was 323, while in 2020 (12 months) it decreased to 266, and in 2021 (five months) it further declined to 233.

**Table 1:** 2019 – 2021 Sexually Transmitted Infections, Out-Patient Department and COVID-19 descriptive data by year.

Variable	2019			2020			2021		
	n (%)	Mean	SD*	n (%)	Mean	SD*	n (%)	Mean	SD*
STI cases	2267 (34%)	323	92	3201 (48%)	266	76	1169 (18%)	233	76
STI cases Males	759 (33%)	111	59	1103 (48%)	90	24	426 (19%)	85	39
STI cases Females	1508 (35%)	218	34	2098 (48%)	177	44	743 (17%)	148	43
OPD First attendance males	42622 (42%)	6088	1245	42582 (42%)	3548	1303	16783 (16%)	3356	925
OPD First attendance females	50359 (40%)	7194	1128	56135 (45%)	4677	1574	19033 (15%)	3806	1165
OPD revisits males	14555 (35%)	2079	730	19138 (45%)	1644	780	8423 (20%)	1684	289
OPD revisits females	15807 (31%)	2258	717	26022 (52%)	2168	1260	8324 (17%)	1664	177
COVID-19 screening	0 (0%)	0	0	6698 (27%)	558	652	18359 (57%)	3671	3079
COVID-19 confirmed	0 (0%)	0	0	914 (65%)	76	126	498 (35%)	99	90

SD* Standard Deviation (Source - Researcher 2022).

Analysed STI data by sex indicated that, on average, 181 females presented to the clinics compared to 95 males during the period from June 2019 to May 2021. The data further revealed that a total of 6,637 STI cases were recorded at OPD during the study period, with 66% (n = 4,349) of cases among female and 34% (n = 2,288) among males, all within the group 15–49 age group.

#### STI, OPD and COVID-19 mean, standard deviation and p-value by sex and period.

The data were further analyzed across three defined periods-pre-COVID-19, first wave, and second wave of the pandemic-as presented in Table 2. Each period spanned an equal number of months. The analysis showed that the mean number of STI cases recorded during the pre-COVID-19 was 329, while the mean dropped to 267 during the first wave and further declined during the second.

**Table 2:** 2019 – 2021 Sexually Transmitted Infections, Out-Patient Department and COVID-19 descriptive data by study period.

Variable	Pre Covid-19				COVID-19 First Wave				COVID-19 Second Wave			
	n (%)	Mean	SD*	*p-Value*	n (%)	Mean	SD*	*p-Value*	n (%)	Mean	SD*	*p-Value*
STI Cases	2634 (40%)	329	86	.232	2141 (32%)	267	65	.899	1862 (28%)	232	79	.434
STI Cases Male	888 (39%)	111	58	<.001*	720 (31%)	90	24	.339	680 (30%)	85	38	.239
STI Cases Females	1746 (40%)	218	34	<.001*	1421 (33%)	177	44	.059	1182 (27%)	147	42	.002*
OPD First Attendance Male above 15	47775 (47%)	5971	1199	<.001*	27472 (27%)	3434	1478	<.001*	26740 (26%)	3342	751	<.001*
OPD First Attendance Female above 15	56995 (45%)	7124	1062	<.001*	35371 (28%)	4421	1782	.001*	33161 (26%)	4145	1045	<.001*
OPD Revisits Male above 15	16973 (40%)	2121	686	<.001*	13254 (31%)	1656	781	.186	12489 (29%)	1561	551	.114
OPD Revisits Female above 15	18628 (37%)	2328	693	<.001*	17826 (36%)	2228	1412	.84	13699 (26%)	1712	631	.222
COVID-19 Screening	0	0	0	1	4816	602	773	.483	20241	2530	2814	.007*
COVID-19 Confirmed	0	0	0	1	873	109	145	.034*	539	67	81	.176

*=p<0.05, SD* = Standard Deviation, (Source - Researcher 2022).

#### STI services during Pre-COVID-19, during and post.

To further investigate the reduction in access to STI services, a segmented regression analysis was conducted using OPD attendance as the proxy dependent variable and the three defined time periods as the independent variables (S4 Data).

When period numerical < 2 (representing the pre-COVID-19 phase), the estimated mean OPD attendance for age group15 – 49 was 13096. This intercept was statistically significant (p < 0.001), indicating a significant baseline attendance level before period numerical reaches 2 (before the COVID-19 period).

When period numerical is between 2 (inclusive) and 3 (exclusive) (COVID-19 period first phase), the estimated mean OPD attendance 15 – 49 is 7855. This intercept is statistically significant (p < 0.001), reflecting a substantial decrease in attendance compared to the pre-pandemic period.

When period numerical > 2, corresponding to the second phase of the COVID-19 pandemic, the estimated mean OPD attendance for the same age group was 7487. This intercept was also statistically significant (p < 0.001), suggesting a further decline in attendance after the first phase of the pandemic.

The segmented regression analysis demonstrated that the relationship between period and OPD attendance among individuals aged 15 – 49 was not constant across different time segments defined by period thresholds. Prior to the COVID-19 pandemic, OPD attendance was estimated to be highest at 13096 a figure that was statistically distinct from the other periods. During the first phase of the pandemic, attendance declined to 7855, a statistically significant decrease compared to both the pre-COVID-19 and second phase periods. In the second phase of the pandemic, attendance further declined to 7487, indicating a continued shift in health-seeking behavior beyond the first phase of the behavior COVID-19 pandemic.

Differences between the three time periods-pre-COVID-19, COVID-19 first wave, and COVID-19 second wave-were examined using Kruskal-Wallis test. The results showed no statistically significant difference in STI cases across the three time periods(*p* = .093). This indicates that, based on the available data, changes in STI cases across the defined timeframes did not result in statistically significant difference in the median number of STI cases.

In contrast, a Kruskal-Wallis test was also conducted to assess the differences in OPD attendance among individuals aged 15 – 49 across the same period. The test revealed a statistically significant difference (X^2 ^= 12.195, *p* = .0022), indicating that attendance varied across the three time periods. Specifically, attendance was higher in the pre-COVID-19 period compared to both phases during the pandemic. These findings suggest that the COVID-19 pandemic had a notable impact on health-seeking behavior, as reflected in OPD attendance patterns, which may have indirectly affected the number of STI cases recorded.

### Qualitative analysis

Following the quantitative data analysis, qualitative data were collected through in-depth interviews with health providers (Clinical Officers). Table 3 presents participant characteristics, indicating that all interviewees had a minimum of five years of work experience in the Out-patient department at their respective health institutions.

**Table 3:** Demographic profile for in-depth interview participants – profile of the participants in the in-depth interviews from the different Facilities.

Facility	Interviewees		Work Experience with STIs
	Male	Female	
Facility A	1	1	> 5 years
Facility B	1	1	> 5 years
Facility C	1	1	> 5 years
Facility D	1	1	> 5 years
Facility E	0	2	> 10 years

From the in-depth interviews with the clinicians and following the processes of data familiarization, coding, and thematic development from the transcribed verbatim interviews, three major themes emerged, as presented in Table 4.

**Table 4:** Emerging Themes and Sub-themes from Transcribed Verbatim – emerging themes from the codes developed from the transcribed verbatims.

Theme	Sub-theme	Direct quotes
Pandemic-induced health behavior change	Stay at home policy	*the directive by Government to the public about avoiding visiting the clinics with minor illnesses*
	Restricted services at the clinic	*several restrictions on all the services offered at the facility*
	Social distancing	*Patients were made to stand far apart to adhere to social distancing*
	Scare to get infected	*it being a clinic this is where you would find a lot of Covid-19 cases so they would be scared to come to the health facility*
Healthcare service delivery strain and disruption	Reduced patient contact time	*we did not really want to keep patients for a long time in the clinic, so we had shortened the screening process*
	Reduced clinicians on duty	*The workforce was affected by the COVID-19 because even the clinicians got infected with COVID-19 and they were given 14 days off*
	Reduced availability of STIs resources	*have not had RPR for Syphilis for an exceptionally long time*
	Scare of COVID-19	*it being a clinic this is where you would find a lot of Covid-19 cases so they would be scared to come to the health facility*
STIs healthcare ecosystem: Availability and limitations	STIs services and products availability	*Operating hours were reduced limiting services and products availability especially at small clinics*
	Restricted services points	*others were on bed rest, off duty, so it affected the number of staff on duty*
	Youth friendly spaces influence	*I know that the youth friendly space was also closed*
	Availability of healthcare providers	*if two clinical officers got infected and I tell you this happened more than once, then you had a serious shortage of manpower*

### Pandemic-induced health behavior change

Access to STI health services and products was affected during the COVID-19 pandemic by a range of factors, including the Ministry of Health’s stay at-home policy, fear of contracting COVID-19 at health facilities, restricted services at the clinics, and social distancing measures. The analysed in-depth interviews through coding and thematic grouping, yielded the theme “Pandemic-induced health behavior change.” This theme captures how access to healthcare services - particularly STI services and products - was disrupted by shifts in health seeking behavior during the pandemic. Interviews with clinicians revealed that many patients were reluctant to visit health facilities due to perceived heightened risk of infection in these settings. One clinician shared:

*“… everyone was scared to come to the facility when they did not have any COVID-19 signs and symptoms. So, for instance if one were having abdominal pains they would stay at home and self-medicate…” (IDI_CO_Female_C_12_2022)*

Another clinician said:

*“I would say yes they were scared of Covid-19, when you move around and it being a clinic this is where you would find a lot of Covid-19 cases so they would be scared to come to the health facility especially if they had STIs because most of the times this is mostly asymptomatic so they will not bother to come to the health facility” (IDI_CO_Female_U_12_2022).*

The Ministry of Health, through a statutory instrument aimed at preventing the spread of COVID-19, issued directives to stay at home, avoid non-essential visits to health facilities, and practice social distancing. These measures directly impacted access to STI health services at the OPD level in clinics. According to one clinician:

*“…several people avoided the clinic especially after the government through the Ministry of Health announced that people should avoid going to the clinics when not extremely sick or spending a long time at the clinic. This really made people to avoid the clinics and the congestions that would put them at risk of getting COVID-19…” (*IDI*_CO_Female_C_12_2022).*

Yet another clinician said:

*“…the directive by Government to the public about avoiding visiting the clinics with minor illnesses. I personally feel the MoH made this statement with the aim of reducing the spread of COVID-19 because the clinic was one area where you could easily get COVID-19 infections. You know our spaces are small…” (IDI_CO_Male_K_1_2023).*

The Ministry of Health directive is echoed further by another clinician who thought:

*“…Ministry of Health announced that people should avoid going to the clinics when not extremely sick or spending a long time at the clinic. This really made people to avoid the clinics and the congestions that would put them at risk…” (IDI_CO_Male_C_12_2022).*

The interviews further revealed that limited access points may have contributed to the reduction in individuals visiting the health facilities. As the pandemic progressed, word spread within the community that several services had been discontinued at health facilities. This assertion was confirmed by a clinician who stated that:

*“…several restrictions on all the services offered at the facility. For instance, family planning services had restrictions on the number of working hours though we still offered the services” (IDI_CO_Female_C_1_2023).*

Several factors contributed to the reduction in the number of individuals seeking healthcare for conditions other than COVID-19, including changes in their health seeking behavior.

### Healthcare service delivery strain and disruption

Healthcare services delivery strain and disruption emerged as a major theme from the interview transcripts. This theme encompassed the challenges experienced in delivering healthcare services due to reduced patient-clinician contact time and limited clinician availability. The most cited barrier to delivery of STI health services and products was the reduction in contact time between patients and clinicians, primarily attributed to fears of COVID-19 transmission. One of the clinicians stated that:

*“…we did not really want to keep patients for a long time in the clinic, so we had shortened the screening process. We could not do all the procedures, so some of the procedures were cut off so that we could shorten the entire process of interaction between us the providers and the patients to avoid patients staying in the clinic for a long time.”* (IDI_CO_Female_E_12_2022).

Another clinician stated that:

*“…if a patient came for COVID-19 related illnesses then there was a chance that they could have been missed for STIs diagnosis because we wanted to have this person attended to very fast to save them from progression and to avoid long times of exposure to an infected person…”* (IDI_CO_Female_D_1_2023).

The disruption in the delivery of resources for STI health services may have been one of the key barriers to providing quality STI health services and products. Clinicians reported that supply of essential resources to various facilities was significantly disrupted.

*“…have not had RPR for Syphilis for an exceptionally long time. Ideally, we could have been confirming diagnosis from here...”* (IDI_CO_Male_C_1_2023).

While another clinician stated that.

*“I know that the supply chain systems were affected across the world, so Zambia was not special or excluded from this interruption…”* (IDI_CO_Male_C_12_2022).

The delivery of STI health services was affected by the supply chain and the healthcare providers shortened time of interaction with the patients.

### STI healthcare ecosystem: availability and limitations

The STI healthcare ecosystem emerged as a central theme, encompassing availability of STI services, products, and youth-friendly spaces, alongside the impact of restricted service points and limitations in peer educator networks. Clinicians reported that the availability of STI services and products was compromised by the absence of healthcare providers, the closure or repurposing of youth friendly spaces, and a lack of resources to conduct confirmatory tests.

The absence of healthcare providers was identified as one of the key factors that hindered the delivery of STI services. Clinicians noted this challenge, stating that:

“*The workforce was affected by the COVID-19 because even the clinicians got infected with COVID-19 and they were given 14 days off. So, for instance if two clinicians were given 14 days off during the same period, it brought about pressure on the remaining clinicians*” (IDI_CO_Female_D_1_2023).

Another clinician stated that:

*“…the people who were frontline workers seeing people who were suffering, and most health workers got infected with Covid-19 also. So, others were on bed rest, off duty, so it affected the number of staff on duty…”* (IDI_CO_Female_E_12_2022).

While another clinician said:

“…*if two clinical officers got infected and I tell you this happened more than once, then you had a serious shortage of manpower…”* (IDI_CO_Male_C_12_2022).

The youth friendly spaces that act as entry points to accessing health services also faced challenges during the COVID-19 pandemic, one clinician stated:

*“To be honest with you, I have seen very few and I do not remember seeing one during the COVID-19 period. I know that the youth friendly space was also closed.”* (IDI_CO_Male_C_1_2023).

## Discussion

The COVID-19 pandemic affected access to STI health services and products, as evidenced by a decline in the number of individuals recorded in the OPD attendance registers. This aligns with similar trends observed in Australia, America, Canada, China, Botswana, and Zimbabwe [9,16–19]. We noted a reduction in OPD attendance across the three periods defined for the purpose of this study – pre-COVID-19, first phase and second phase of the COVID-19 pandemic – with the pre-COVID-19 period representing the assumed baseline for normal service utilization. The analyzed data indicated a moderately strong positive relationship between OPD attendance and reported STI cases. Increase in OPD attendance were associated with corresponding increases in the number of STI cases reported. Conversely, the reduction in STI cases during the pandemic may imply that a significant number of STI cases went undiagnosed and untreated during this period.

The Zambia Demographic Health Survey (ZDHS) of 2018 [9] reported that males were more likely than females to present at the health facilities for STIs treatment. However, in our study, we found that during the COVID-19 pandemic, females were two times more likely to present at the clinic with STIs for treatment compared to male’s contrary to the ZDHS report. This shift may have been influenced by the mask-wearing and social distancing measures, which created a sense of anonymity and reduced perceived judgement from clinicians, thereby encouraging more women to seek STI treatment.

The observed reduction in OPD attendance during the pandemic suggests that individuals with conditions other than COVID-19 or common colds may have avoided visiting health facilities. These findings are consistent with results from other studies [12,13,17,19–23], which also reported a decline in OPD attendance and a corresponding decrease in STI cases. This raises important questions about what may have happened to STI cases that went unreported or untreated during the pandemic period.

Our findings indicate that the mean attendance declined from 329 during the pre-COVID-19 period to 267 and 232 in the first and second phase of the COVID-19 period, respectively. This trend is consistent with the findings of a cluster randomized trial in Zambia, which assessed the impact of COVID-19 on access to sexual reproductive health, Phiri et al. [22] reported a reduction in access to SRH services during the pandemic period.

Analysed data from the OPD registers also revealed a decline in STI cases, a trend corroborated by qualitative findings from in-depth interviews with clinicians, who noted reduced number of patients presenting with STI-related complaints. These findings align with another Zambian study that utilized socio-economic impact assessment survey data to examine the effects of the COVID-19 pandemic on the utilisation of sexual and reproductive health services. This study also reported a decline in access to family planning and sexual and reproductive health services [22].

Our study established that individuals with health conditions other than COVID-19 were reluctant to visit the health facilities due to fear of contracting the virus, as reported by clinicians during in-depth interviews. The in-depth interviews further revealed that this fear was shared by healthcare providers, who expressed concern about their own risk of infection. These findings are consistent with a study in India by Bhargava [21], which reported that fear of visiting health facilities among patients led to reduced detection of infections other than COVID-19. Similarly, a study conducted in Spain concluded that the decline in sexually transmitted infections was largely attributable to reduced hospital visits, driven by fear of COVID-19 infection [21].

Our study established that restrictions on movement and access to health facilities during the COVID-19 pandemic may have limited access to healthcare for individuals with conditions other than COVID-19. These findings are consistent with those of a study conducted in Italy by Cusini et al. [24], which postulated that lockdown measures contributed to a reduction in non-acute STI cases reported at the health facilities. Similarly, a cross-sectional study conducted in China during the lockdown observed a 22% decrease in sexual desire, a 44% decrease in the number of sexual partners, and widespread disruption in access to health facilities [25,26]. In another cross-sectional study, Mambo et al [27] reported a decline in access to sexual and reproductive health services during the lockdown period, with 68.9% of respondents citing lack of transportation as the primary reason for their inability to travel between locations.

Findings from our study further revealed that the Ministry of Helth’s stay-at-home policy influenced the health-seeking behavior of patients. An emerging theme from the clinician interviews was “pandemic-induced health seeking behavior change” which manifested as a reduction in OPD attendance, driven by multiple several factors. These results are consistent with those of a scoping review conducted by VanBenschoten et al. [28], which reported reduced access to – and reduced utilisation of – sexual and reproductive health services during the COVID-19 pandemic. These reductions were closely linked to policy directives such as lockdowns and stay-at-home orders, which discouraged individuals from visiting health facilities and heightened public fear of exposure to infection in those settings.

The reduction in OPD attendance may, in the long-term, lead to increased STI-related complications and a rise in new HIV infections. This assumption is supported by a modelling study conducted by Hogan et al. [29], which projected an increased in HIV infections in high-burden regions as a result of disruptions in access to health services and products. Further support is provided by Bonett et al. [30], who proposed that undiagnosed STI cases during the pandemic may have contributed to higher rates of infections and complications post the COVID-19 period.

Our study attributed the reduction in STI cases seen at OPD to limited availability of providers for STI, family planning, and maternal and child health services, as well as to reduced service hours implemented to minimize health providers’ exposure to COVID-19. These service delivery changes were grouped under the emerging theme of “healthcare service delivery strain and disruption,” as identified in clinician interviews. Indeed, healthcare providers shifted their focus towards COVID-19 related services, often at the expense of patients with other health conditions, thereby increasing their vulnerability to delays in accessing treatment, care, and support services.

Our study found that healthcare providers reduced the amount of time spent with patients and avoided procedures involving direct physical contact, primarily due to fear of COVID-19 infection. These adjustments negatively affected the quality of STI screening provided, aligning with the findings from a study conducted in New York, which reported that reduced provider availability led to decreased diagnosis of asymptomatic infections such as chlamydia in health facilities [31]. These results correspond to Levesque’s [32] dimension of “accommodation,” which highlights service hours and appointment availability as critical determinants of healthcare access.

Furthermore, our study demonstrates that operational changes among healthcare providers revealed systemic challenges in ensuring the availability and accommodation of STI services for health seekers. This is consistent with findings from studies conducted in the African region, Asia, the United States, and Europe, which reported that, during the COVID-19 pandemic, STI and family planning services were often discontinued or reduced due to a shift in focus toward COVID-19-related care [30,31]. Tao et al. [33] similarly found that access to and delivery of STI health services were hindered by precautionary measures intended to protect healthcare workers and prevent intra-transmission of COVID-19, resulting in reduced service availability.

Our study revealed that reduced operating hours and patient appointments during the COVID-19 pandemic led to limited access to STI health services. This finding aligns with the third theme that emerged from the clinician interviews - “STIs healthcare ecosystem: availability and limitations”- which highlighted diminished availability of healthcare providers and a reduction in service delivery points. Levesque’s conceptual framework emphasizes both the abilities of the health seekers and their capacity to reach healthcare facilities as critical components of access.

During the pandemic, individuals seeking STI services were adversely affected by restricted access to health facilities. This observation is supported by studies conducted in Australia, the United States, and Italy, which reported decreased STIs health seeking behavior among patients during the COVID-19 period [31,34,35]. The inability for patients to access health services was largely a consequence of public health policies aimed at reducing COVID-19 transmission, which had the unintended effect of further restricting access to STI services, as noted by other researchers [36–39].

## Conclusion

The access, availability, and delivery of STI health services and products were compromised by a combination of factors. The alignment of these findings with Levesque’s “accommodation” dimension underscores the critical role that operating hours and patient appointments play in shaping healthcare access.

Restrictions such as stay-at-home mandates, fear of infection, lockdowns, and logistical challenges impended individuals’ ability to seek care. These barriers are consistent with Levesque’s framework, illustrating the complex relationship between policy, healthcare infrastructure, and individual health-seeking behaviors.

The COVID-19 pandemic exposed systemic vulnerabilities in healthcare delivery when confronted with unexpected crises. To mitigate future disruptions, healthcare systems should consider adopting flexible service delivery models. The widespread policy misinformation observed during the pandemic highlights the need for more effective health communication strategies. Policymakers and healthcare institutions must also invest in research and preparedness measures to better understand and respond to public health emergencies on healthcare access, availability, and delivery.

## Recommendations

To address the effects of the COVID-19 pandemic on the accessibility, availability, and delivery of STI health services and products among young adults in Lusaka, several recommendations were drawn that may inform handling of future pandemics.

Enhance Health Communication and Education **-** Given the role of public health directives in influencing health-seeking behavior, it is crucial to improve health communication strategies during pandemics.

Flexible Service Delivery Models - To mitigate disruptions, healthcare facilities should consider adopting flexible service delivery models. This study through the interaction with the healthcare providers has proposed an anonymous online STIs screening flow chat that could be developed and adopted to eliminate the challenges that young adults and older people face when they suspect STI infections.

Proposed anonymous online STI screening (Source - Researcher 2023)

Adaptation of Healthcare Infrastructure - Health facilities should work on balancing pandemic care with the provision of other essential services, such as STI health services. This might require revisiting healthcare infrastructure and resource allocation strategies.

## Supporting information

S1 Data In-depth Interview Guide – in-depth interview guide, general questions used in the interviews when collecting qualitative data from the clinicians. (PDF)

S2 Data Qualitative Data Codes - qualitative data codes generated from the transcribed verbatims from the clinicians. (PDF)

S3 Data Anonymized Out-Patient Data – anonymized Out-patient department (OPD) attendance quantitative data from 2019 to 2021 used for all descriptive and statistical analysis. (XLSX)

S4 Data Segmented Regression Analysis - output of data analysis from the segmented regression analysis (Piecewise Regression). (PDF)
