# Supplementary material for: Health crisis within a crisis: Effect of COVID-19 on STI services for young adults in Lusaka, Zambia
Source: PLOS Glob Public Health. 2025 Jul 3;5(7):e0004891. doi: 10.1371/journal.pgph.0004891 (PMC12225784; doi:10.1371/journal.pgph.0004891)
Supplement: S1 Data — (PDF) [file pgph.0004891.s001.pdf]

Data Collection – In-depth Interviews

The purpose of this study is to establish the effect of the COVID-19 pandemic on access, availability, and delivery of STI health services and products to young people in Chelstone. The study will inform health systems on how to handle future pandemics in relation to access to STI health services and products.

In order to understand the challenges that the health systems might have faced, I will conduct one-on-one in-depth interviews with clinicians attending to STI cases at Clinics in Chelstone Sub-district. Indeed, what I learn from the interviews will be analysed and provide evidence for health systems access and delivery improvement in pandemic situations.

You are being asked to be in this study because you a clinician at the target clinics who worked during the COVID-19 and pre COVID-19 in Chelstone Sub-district and you are an expert who can provide your opinion on the problem including giving potential solutions.

Thank you very much for taking time to participate in this study today.

Demographics

Male / Female

How long have you worked at this facility?

How long have you worked in OPD?

Did you work in OPD during and before COVID-19 pandemic?

Question 1: To establish the effect of COVID-19 on the delivery of STI health services and products to young people (15 – 49 years) in Lusaka – Qualitative data to be collected through in-depth interviews.

In your opinion, how did COVID-19 pandemic affect the delivery of STI health services and products to young people?

Follow up question – In your opinion delivery of STI health services and products is multifaceted, what facets were affected by COVID-19?

Probe: Any sections or departments that were closed or had reduced manpower?

Did you face any challenges with testing reagents?

Before the pandemic what kind of STI health services and products were offered at the Clinic?

Follow up question – Do you know of any youth friendly spaces in this area and the services they offer?

Probe: Did the youth friendly spaces refer anyone for STI screening before the pandemic?

What about during the pandemic?

In your view, how did the COVID-19 pandemic affect access to STI health services and products by young people at this Clinic?

Follow up question – in your view what else affected access to STI health services and products by young people?

Probe: Do young people present with STI cases at this facility?

Did the numbers of those presenting with STI cases change in anyway?

What solutions would you propose in future pandemics to allow you deliver STI services better?

Question 2: To assess the effect of COVID-19 pandemic on availability of STI screening services to young people in Lusaka – Qualitative data to be collected through in-depth interviews.

In your view, how did the COVID-19 pandemic affect the availability of STI health services at the clinic and/or youth friendly spaces?

Follow up question – in your view what sexual reproductive health services were affected by availability or non-availability of STI health services and products?

Probe: do you think the availability of healthcare providers change during the pandemic?

In your judgement, how available were STI screening services to young people at the Clinic?

Probe: What services were available?

In your opinion, did the number of STI cases screening increase in Outpatient Department during the COVID-19 pandemic?

Follow up question – If the STI cases increased or decreased, why did this happen in your opinion?

Probe: How available were Lab results or testes during the pandemic?

Did the turn around time change for STI samples that needed confirmation?

What solutions would you propose in future pandemics to allow you make STI services available without disruption?

Thank you very much for your time.
