# Supplementary material for: Health crisis within a crisis: Effect of COVID-19 on STI services for young adults in Lusaka, Zambia
Source: PLOS Glob Public Health. 2025 Jul 3;5(7):e0004891. doi: 10.1371/journal.pgph.0004891 (PMC12225784; doi:10.1371/journal.pgph.0004891)
Supplement: S2 Data — (PDF) [file pgph.0004891.s002.pdf]

Data Codes

A case study of Chelstone.

Specific Objectives

- To investigate the effect of COVID-19 pandemic on access to STIs health services and products to young people in Lusaka.
- To establish the effect of COVID-19 on the delivery of STIs health services and products to young people in Lusaka.
- To assess the effect of COVID-19 pandemic on availability of STIs screening services to young people in Lusaka.

**Code Name**	**Code Description**
**STIs services delivery**	
Delivery of STIs services	Use when clinician talks about how STIs services were offered during the COVID-19 period and before at the facility
Stay at home policy	Use when clinician talks about the COVID-19 movement restrictions imposed by the Ministry of Health
Scared of COVID-19	Use when the clinician talks about people avoiding coming to the clinic because of COVID-19
Reduced patient contact time	Use when the clinician talks about minimal patient screening time to avoid extended exposure to COVID-19
Reduced number of clinicians on duty	Use when the clinician talks about health workers getting sick, on leave or given days off due to suspected COVID-19 infection
**STIs services and products availability**	
STIs service and products availability	Use when the clinician describes the availability of STIs services and products during the COVID-19 period at the facility
Youth friendly spaces influence	Use when the clinician describes the role of youth friendly spaces in referring STI cases to OPD
Peer educator network availability	Use when the clinician talks about the availability of peer educators during the COVID-19 period
Restricted services points	Use when the clinician talks about the closure of services points at the facility (e.g., MCH, HTS)
Availability of health providers	Use when clinicians talk about the number of health workers on duty in OPD during the COVID-19 period
**Access to STIs services and products**	
Access to STIs services	Use when clinician talks about how patients were accessing STIs services and products during the COVID-19 period
OPD STI cases	Use when the clinician talks about the increased/reduced number of STI cases seen in OPD before and during the COVID-19 period
General OPD attendance	Use when the clinician describes the general OPD attendance by patients before and during the COVID-19 period
Reduced STI cases	Use when clinician refers to reduced confirmed STI cases seen during the COVID-19 period in OPD
**General COVID-19 effects**	
Reduced sexual contact	Use when the clinician describes the reduced sexual contact during COVID-19 period
Increased sexual contact	Use when the clinician describes the increased sexual contact during the COVID-19 period

High risky behaviour	Use when clinician describes the high-risk behaviour by young people during the closure of schools
Alternative STI access methods	Use when clinician talks about other ways of accessing or delivering STI services and products to young people

Themes and subthemes from the codes

Theme	Sub-theme
Pandemic -induced health behaviour change	Stay at home policy
	Restricted services at the clinic
	Social distancing
	Scare to get infected

Theme	Sub-theme
Healthcare service delivery strain and disruption	Reduced patient contact time
	Reduced clinicians on duty
	Scare of COVID-19

Theme	Sub-theme
STIs healthcare ecosystem: Availability and limitations	STIs services and products availability
	Restricted services points
	Youth friendly spaces influence
	Peer educator network availability
