# Supplementary material for: Health crisis within a crisis: Effect of COVID-19 on STI services for young adults in Lusaka, Zambia
Source: PLOS Glob Public Health. 2025 Jul 3;5(7):e0004891. doi: 10.1371/journal.pgph.0004891 (PMC12225784; doi:10.1371/journal.pgph.0004891)
Supplement: S4 Data — (PDF) [file pgph.0004891.s004.pdf]

```
. reg OPDAttendance1549 if PeriodNumerical <2

      Source |       SS           df       MS      Number of obs   =         8
-------------+----------------------------------   F(0, 7)         =      0.00
       Model |           0         0           .   Prob > F        =         .
    Residual |  35385683.5         7  5055097.64   R-squared       =    0.0000
-------------+----------------------------------   Adj R-squared   =    0.0000
       Total |  35385683.5         7  5055097.64   Root MSE        =    2248.4

------------------------------------------------------------------------------
OPDAtte~1549 |      Coef.   Std. Err.      t    P>|t|     [95% Conf. Interval]
-------------+----------------------------------------------------------------
       _cons |   13096.25   794.9133    16.48   0.000     11216.58    14975.92
------------------------------------------------------------------------------

. reg OPDAttendance1549 if PeriodNumerical >= 2 & PeriodNumerical < 3

      Source |       SS           df       MS      Number of obs   =         8
-------------+----------------------------------   F(0, 7)         =      0.00
       Model |           0         0           .   Prob > F        =         .
    Residual |  74193865.9         7  10599123.7   R-squared       =    0.0000
-------------+----------------------------------   Adj R-squared   =    0.0000
       Total |  74193865.9         7  10599123.7   Root MSE        =    3255.6

------------------------------------------------------------------------------
OPDAtte~1549 |      Coef.   Std. Err.      t    P>|t|     [95% Conf. Interval]
-------------+----------------------------------------------------------------
       _cons |   7855.375   1151.039     6.82   0.000     5133.601    10577.15
------------------------------------------------------------------------------

. reg OPDAttendance1549 if PeriodNumerical >2

      Source |       SS           df       MS      Number of obs   =         8
-------------+----------------------------------   F(0, 7)         =      0.00
       Model |           0         0           .   Prob > F        =         .
    Residual |  20515787.9         7  2930826.84   R-squared       =    0.0000
-------------+----------------------------------   Adj R-squared   =    0.0000
       Total |  20515787.9         7  2930826.84   Root MSE        =      1712

------------------------------------------------------------------------------
OPDAtte~1549 |      Coef.   Std. Err.      t    P>|t|     [95% Conf. Interval]
-------------+----------------------------------------------------------------
       _cons |   7487.625   605.2713    12.37   0.000     6056.386    8918.864
------------------------------------------------------------------------------
```
